# Develop an efficient inoculation technique for *Fusarium solani* isolate “TJP-2178-10” pathogeny assessment in *Phalaenopsis* orchids

**DOI:** 10.1186/s40529-021-00310-z

**Published:** 2021-03-31

**Authors:** Shu-Yun Chen, Yan-Jeng Wu, Ting-Fang Hsieh, Jiunn-Feng Su, Wei-Chiang Shen, Yung-Hsiang Lai, Pen-Chih Lai, Wen-Huei Chen, Hong-Hwa Chen

**Affiliations:** 1 Department of Life Sciences, National Cheng Kung University, 701 Tainan, Taiwan; 2 Plant Pathology Division, Taiwan Agricultural Research Institute Council of Agriculture, 413008 Taichung, Taiwan; 3 Department of Plant Pathology and Microbiology, National Taiwan University, 10617 Taipei, Taiwan; 4 Taida Horticultural Co., Ltd, Da Tsun County, 31306 Chang Hwa, Taiwan; 5 Orchid Research and Development Center, National Cheng Kung University, 701 Tainan, Taiwan

**Keywords:** *Fusarium* spp., *Phalaenopsis*, Phenotyping, Symptom, Yellow leaf disease

## Abstract

**Background:**

*Phalaenopsis* is one of the important ornamental plants worldwide. It plays the most significant role in flower exportation in Taiwan. However, the yellow leaf disease caused by *Fusarium* spp. has reduced the orchid flower yield 10–50 % yearly. Varieties resistant to yellow leaf disease associated with *Fusarium* is urgently needed for orchid growers and breeders, and is the ultimate solution for the long-term goal. To achieve this, phenotyping is the first step and the most necessary information for further studies, such as resistance gene identification, quantitative trait loci identification, and genome-wide association study.

**Results:**

The inoculation of *Fusarium* was performed in either abbreviated stem or detached leaf, and the pros and cons were compared. The former is the general method of phenotyping for estimating the tolerance to yellow leaf disease of *Phalaenopsis*, but it is time-consuming and spacy, and thus not suitable for the assessment of large numbers of samples. In contrast, the latter not only showed a similar trend of disease severity with time reduced to only one fourth of the former one but also less space needed.

**Conclusions:**

This solution allows a better phenotyping approach for the fast detection of yellow leaf disease associated with *Fusarium* in a large number of *Phalaenopsis* samples.

## Background

Orchids are important flowers for horticultural production and exportation worldwide. Among them, *Phalaenopsis* app. are the most popular and significant orchids. In Taiwan, the export value of *Phalaenopsis* in 2019 was about 140 million US dollars which covered 75 % of the total value of orchid exportation (https://www.coa.gov.tw/).*Fusarium* spp., pathogens damage crop widely and cause up to 50 % yield loss in soybean, banana, tomato, and wheat (Perincherry et al. 2019). For orchids, symptoms developed after infection by *F. solani* include leaf spots, leaf blight, sheath rot, and root rot. The infection spread worldwide because of international trade and monoculture (Swett and Uchida 2015).

*Fusarium solani* with host specificity of *Phalaenopsis* has been found in Taiwan (Chung et al. 2011), Korea (Kim et al. 2002), Hawaii (Swett and Uchida 2015), and Australia (Laurence et al. 2016). Infection by *F. solani* causes a reduction of 20–30 % yield every year in Taiwan (Su et al. 2010). To suppress the activity of *Fusarium*, 16 fungicides have been tested for growth inhibition of *F. solani*. However, although the fungicides showed effect to inhibit the mycelial growth of *Fusarium* (Su et al. 2014), none of them show significant effects on the reduction of the damage caused by *F. solani* in the greenhouse environment (Su et al. 2014), or after a long distance of shipment from country to country for several months (Liao et al. 2012). Instead of chemical treatment to reduce the infection rate of *Fusarium* on *Phalaenopsis*, development of resistant varieties with quantitative treat loci (QTLs) resistance to *F. solani* via breeding program will be an alternative solution. The resistance genes or QTLs to *Fusarium* spp. have been identified from Arabidopsis (Diener and Ausubel 2005), wheat (Buerstmayr et al. 2002) and pea (Coyne et al. 2015). However, so far no reports of *Fusarium* resistance QTL have been reported for orchids. Phenotyping symptoms after pathogen inoculation is the most important step for the identification of resistance genes or QTLs. A long duration of 4–6 weeks for disease progression after inoculation at the abbreviated stem of live *Phalaenopsis* plants have hampered the assessment. Even worse is that a large space is needed for examination enough plants for *Phalaenopsis-Fusarium* study (Su et al. 2012).

In this study, a modified method for assessment of the yellow leaf symptom infected by *F. solani* in detached leaf was established with reduced observation time of 6 days and much less space needed. In addition, the present study shows that similar trends of symptom development were observed between the infection of *F. solani* in the detached leaf and that in the abbreviated stem. Furthermore, symptom detection based on the detached leave method was stable and feasible for examining a large quantity of plants.

## Materials and methods

## Sample collection

Five *Phalaenopsis* cultivars including TAI_A2945, TAI_A7403, TAI_A9168, TAI_A10040, TAI_A10746 from Taida Orchid Nursery were used in this research, to fit the real transportation situation, twenty mericlones cultivated were provided from orchid nursery, each individual was planted in 2.5-inch pots for 2–3 months which close to the true plant size for oversea production. Twenty individuals of each cultivar were used in each replicate and separated equally for *Fusarium* inoculation to detached leaf and abbreviated stem. For each tested tissue, 7 individuals were infected with *Fusarium*, and 3 individuals were inoculated with water as control. For each variety, a total of 60 plants were used, including 10 plants per tissue per variety were used in one replicate, and repeated 3 times independently. Three replicates in total were performed with one-week interval among each replicate. Before the inoculation, the 2nd or 3rd leaf from the top was collected as a mature leaf based on the comparison of leaf length between 1st and 2nd leaf from the top. When the length of the 1st leaf shorter than half of the 2nd leaf, the 3rd leaf was represented as mature leaf. On the other hand, when the length of the 1st leaf longer than half of the 2nd leaf, the 2nd leaf was collected as matured leaf. The detached leaf was prepared from one mature leaf of each individual which cut around 8.5 cm from the apexes and then fixed the detached leaf in a 15 cm diameter dish. We kept all plant materials in a room with the constant temperature at 27 °C for two days before the detached step to make sure all plants started with the same environment.

### *Fusarium* inoculation

The procedures of inoculation for two organs were performed as previously described by Su et al. (2012). Briefly, seven wounds arranged in a circle was created before inoculation. The pathogenic fungi, *Fusarium solani*, was provided from Taiwan Agricultural Research Institute isolated from the orchid nursery in Taiwan. The spore suspension was diluted to 10^3^ pfu/ml for detached leaf inoculation and 10^5^ pfu/ml for the abbreviated stem inoculation. The seven wounds in a whorl was inoculated with 250 µl inoculum of spore suspension at once and covered with micropore (3 M) to maintain the liquid which was then removed 2 days after inoculation. The infected detached leaves, and plants infected at abbreviated stem were incubated in the climate control room (HiPoint, EH-1800) set at 27 ± 1℃ with 100 % humidity. Photos of each infected detached samples were taken from the 2nd day to the 6th day after inoculation. For plants infected in abbreviated stem, photos were taken from the 2nd day after inoculation to the symptom rank 9 shown in any tested cultivars. The isolate of *F. solani* we used to inoculate plants was “TJP-2178-10”, which showed the most severe symptom on 28 days post inoculation (dpi) (data not shown).

## Symptom ranking

To distinguish the degree of pathogen resistance from all inoculated detached leaves or abbreviated stems, the disease severity level (DSL) was ranked each day after inoculation for 6 days (detached leaf) or for more than 3 weeks (abbreviated stem). Disease severity index (DSI) is a percentage calculated by the following formula. The number of individuals of each DSL level times each DSL, and make a summation of all different DSL. The sum is then divided by the score of the total levels of DSL times total number of individuals in the same variety (Chiang et al. 2017).

$$DSI\left(\%\right)=\frac{\sum(DSL\times number\;of\;individuals\;in\;this\;level\;of\;variety)}{Tatal\;level\;of\;DSL\;times\;total\;number\;of\;individuals\;in\;the\;same\;variety}$$

For instance, we have 7 individuals in total 11 ranks of DSL, 3 of them ranked as DSL 3 and the rest of 4 ranked as DSL 4, then the DSI here will be calculated as following: (3*3 + 4*4)/(11*7)*100 %=32.4 %.

## Results

### Determination of DSL ranks for the infected detached leaf and infected plant at abbreviated stem

**Fig. 1:**
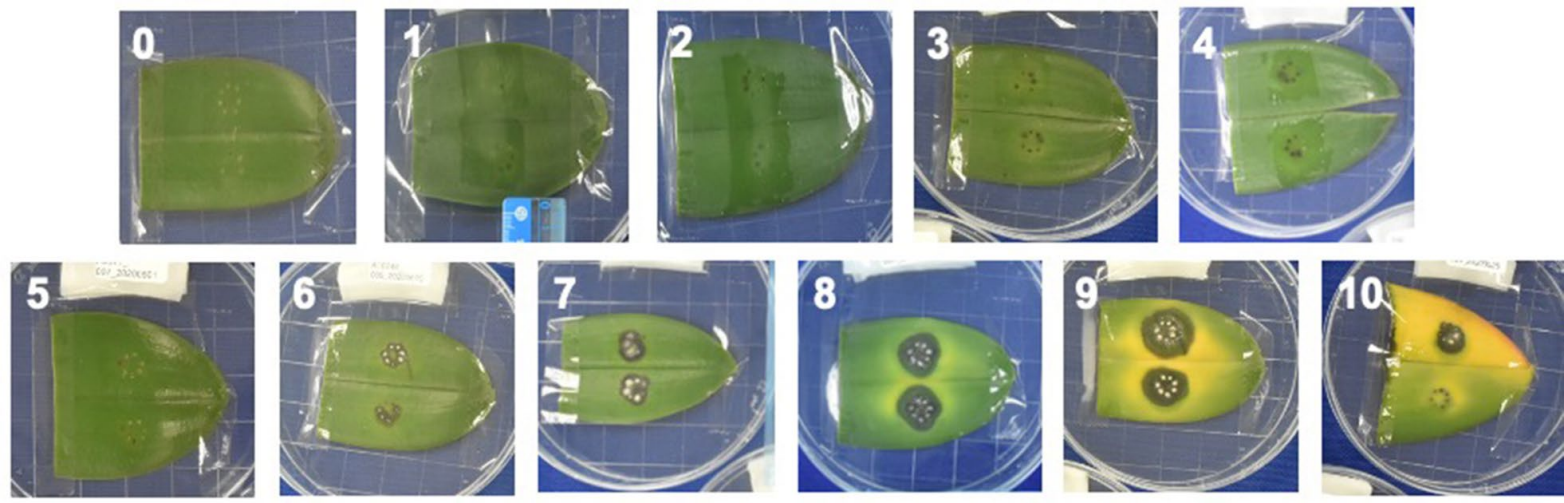
Severity rank of detached leaf. (0): no symptom; (1) Number of black holes less than 3; (2) Number of black holes between 4–7; (3) Black holes connected together; (4) Black spread outside of the wound; (5) Less than 3 holes filled by hypha; (6) Number of holes filled by hypha between 4 to 7; (7) The hypha mixed together; (8) Yellow symptom started; (9) Yellow symptom fill 50 % of the leaf area (10) The red symptom showed

**Fig. 2:**
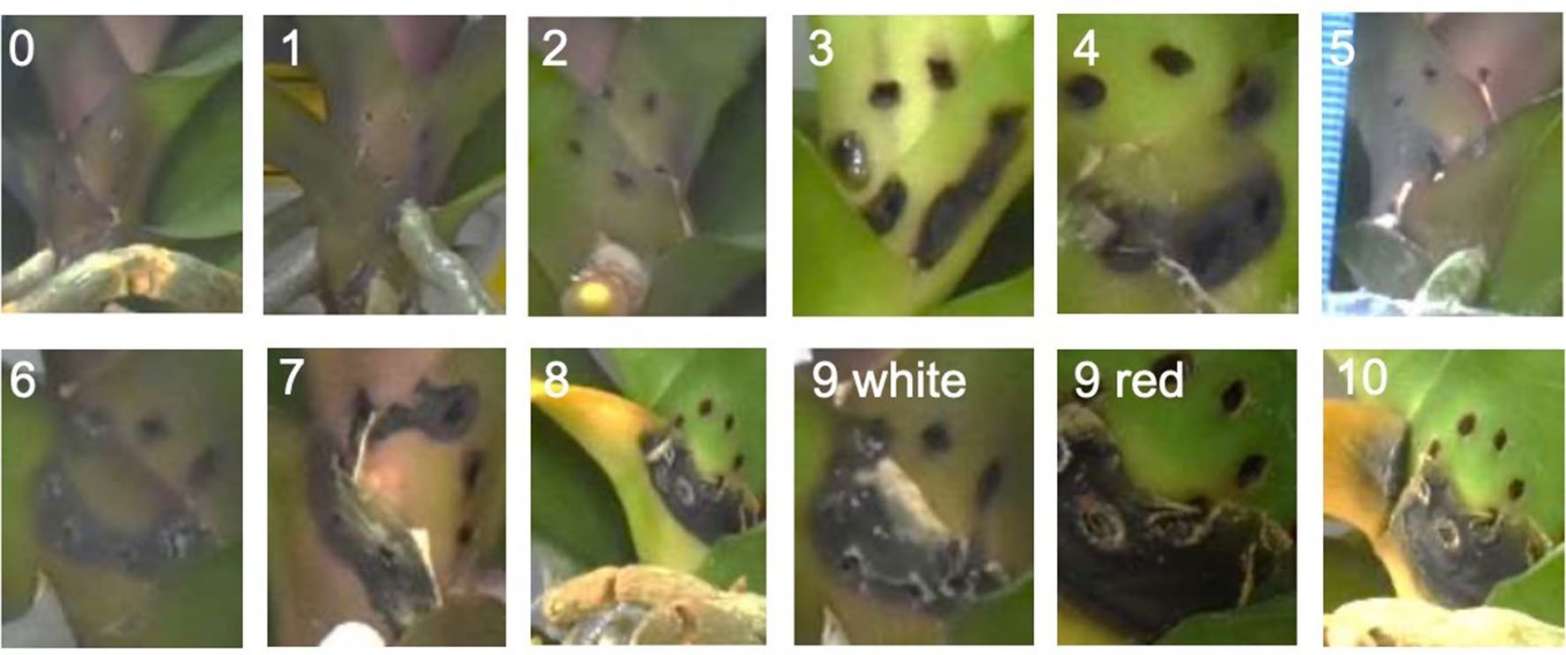
Severity rank of abbreviated stem. (0): No black holes; (1) Number of black holes less than 3; (2 )Number of black holes between 4–7; (3) Black holes connected together; (4) Black spread outside of the wound; (5) Less than 3 holes filled by hypha; (6) Number of holes filled by hypha between 4 to 7; (7) A region of white area showed; (8) Yellow symptom showed; (9) White / Red spots appeared; (10) Yellow leaf broke

A pre-examination was performed to distinguish the different ranks of DSL by inoculating *Fusarium* on the detached leaf or at the abbreviated stem. Variety TAI_A10746 was selected to show the scale of DSL, except the rank 10 of detached leaf (variety CH151, Fig. 1). The DSL was ranked as followed: Level 0: no symptom; level 1: number of black holes less than 3; level 2: number of black holes between 4 and 7; level 3: black holes connect together; level 4: black lesion spreads outside of the wound; level 5: less than 3 holes filled by the hypha from *F. solani*; level 6: 4 to 7 holes filled by hypha from *F. solani*; level 7: the hypha mixed together; level 8: yellow symptom emerges; level 9: yellow symptom diffuses to an entire detached leaf or abbreviated stem; level 10: the red spores show (Figs. 1 and 2). These DSL levels were applied to the photos taken from all five varieties, and three replicates for calculating the DSI of different tissues.

### Similar trends of symptom development from detached leaf and abbreviated stem inoculated with *F. solani*

**Fig. 3:**
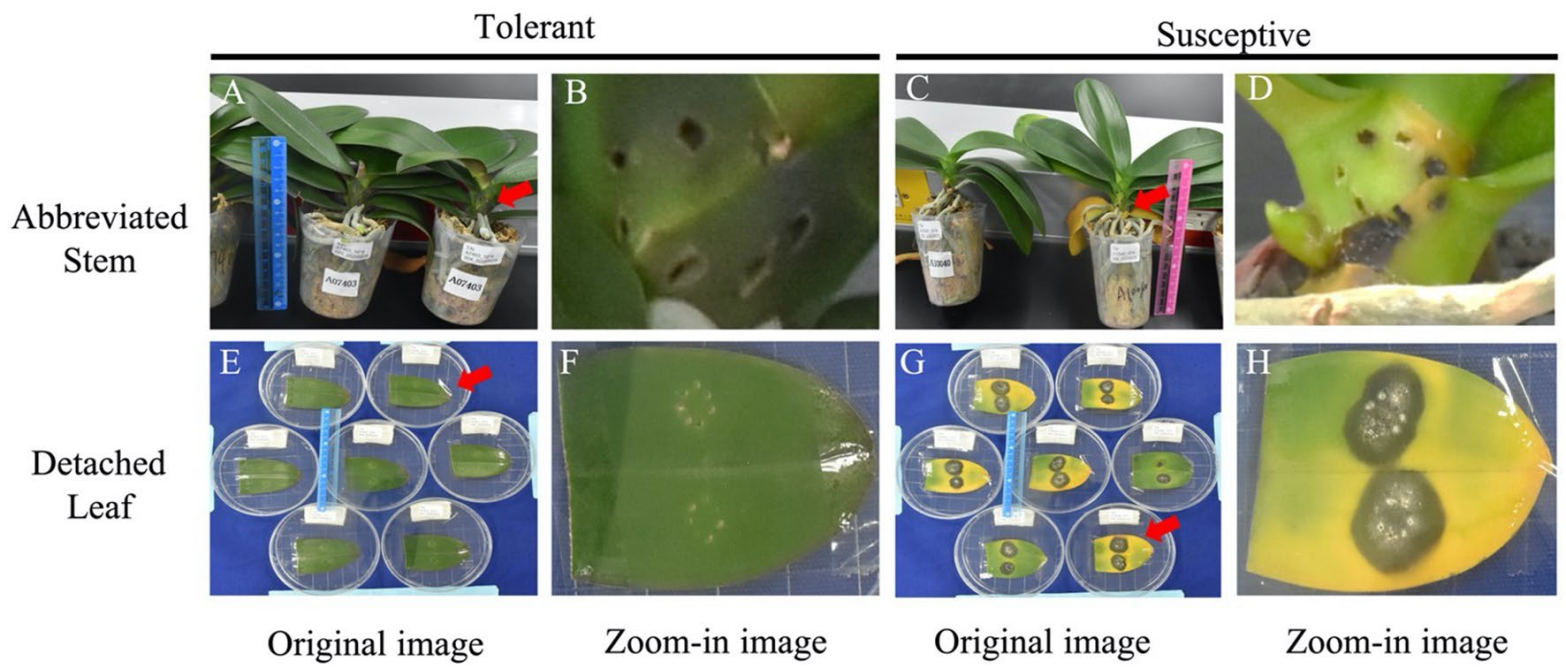
The phenotypes of tolerant and susceptive cultivars developed on abbreviated stem or detached leaf. The original (**a**, **c**, **e**, **g**) and zoom-in (**b**, **d**, **f**, **h**) images were shown from abbreviated stem and detached leaf respectively. Varieties TAI_A7403 and TAI_A10040 were shown as tolerant cultivar and susceptive cultivar respectively

**Fig. 4:**
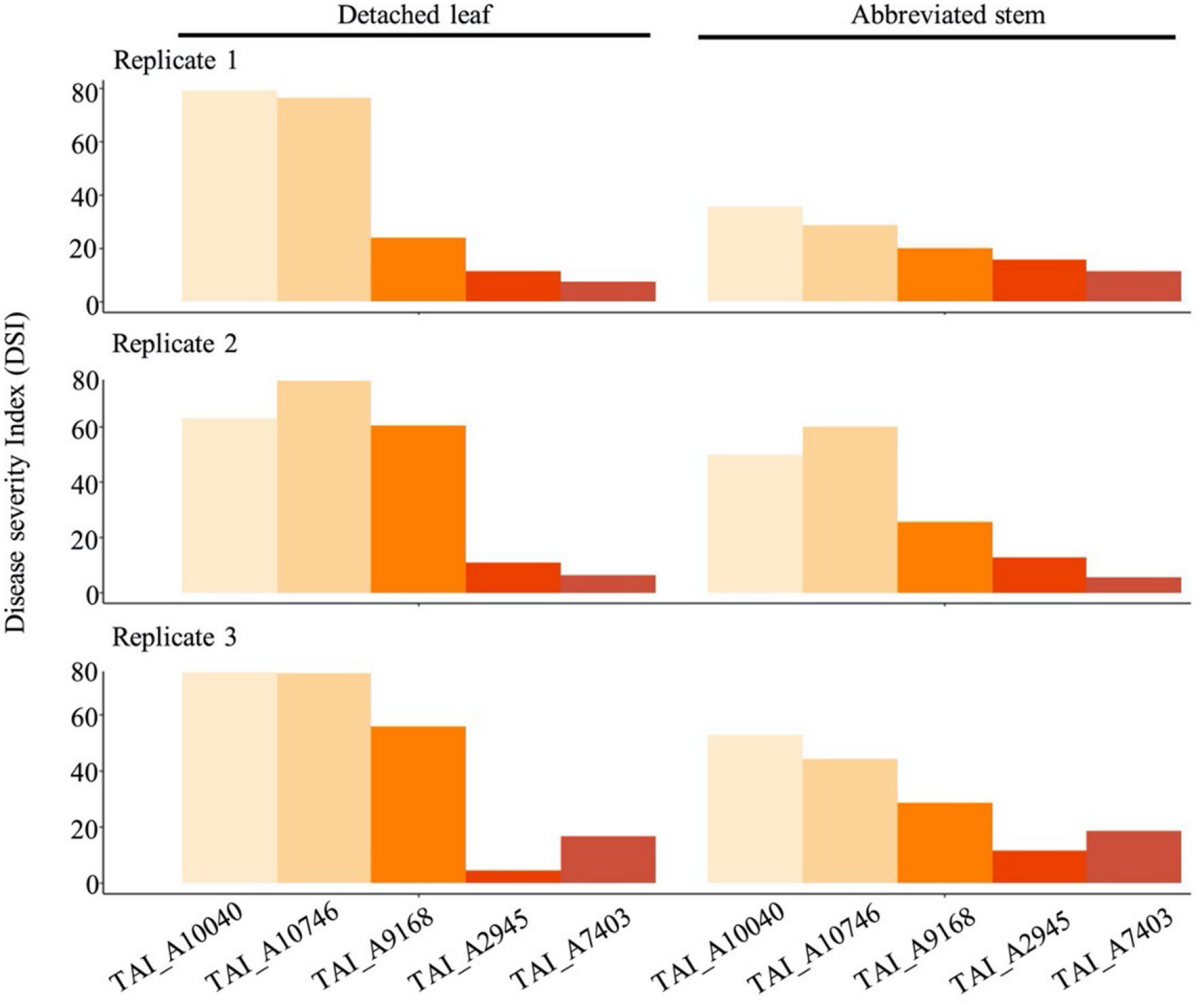
The comparison of DSI among replicates based on organs

According to previous test, cultivar TAI_A7403 and TAI_A10040 were highly tolerant and susceptible to *F. solani* infection. We first checked whether the symptoms on detached leaf and abbreviated stem would be similar after inoculated with *F. solani*. First of all, the symptoms of tolerant and susceptive cultivars that developed on the detached leaf and abbreviated stem were shown. There were no symptoms in the detached leaf and abbreviated stem of variety TAI_A7403 (Fig. 3a, b, e, f). In contrast, strong yellow leaf symptoms in both the detached leaf and the abbreviated stem were revealed for variety TAI_A10040 (Fig. 3c, d, g, h). Second, similar disease severity index patterns were recorded among five tested varieties between detached leaf and abbreviated stem (Fig. 4). Highly positive correlations were detected between the infected detached leaf and the infected plants at abbreviated stem among replicates, as the R square was 0.96, 0.91, and 0.95 for replicate 1, 2, 3, respectively, and p-value lower than 0.05 of all replicates (Table 1).

**Table 1:** The correlation of each replicate between abbreviated stem and detached leaf

	Replicate 1	Replicate 2	Replicate 3
r	0.96	0.91	0.95
*p* value	0.0099	0.0309	0.0125

### Stability of detection and reduction of the observation period by infection in the detached leaf

The stability of DSI among replicates developed on each organ is crucial for the evaluation of the reliability of the inoculation technique. The DSIs for both infected detached leaf and infected plants at abbreviated stem showed significant highly correlated among all replicates (Table 2), suggesting that tested varieties showed consistent yellow leaf symptoms from these organs, and both infections at detached leaf and at abbreviated stem are reliable inoculation techniques. The detached leaf has the highest correlation coefficient 0.96 between replicate 2 and 3, and the lowest correlation coefficient 0.84 between replicate 1 and 2 with the p-value 0.0081 and 0.0771 respectively. In contrast, the correlation among replicates from the abbreviated stem was higher than 0.9 with p-value 0.0292 (replicate 1 vs. replicate 2), 0.0132 (replicate 1 vs. replicate 3), and 0.0299 (replicate 2 vs. replicate 3) (Table 2). In addition, symptom observation time is also an essential point to be considered before screening a large number of samples or varieties. In this study, it took about 20 days for abbreviated stem (ranged from 21 to 32 days), while it took less than one week (6 days) for the detached leaf to differentiate the DSI among orchid varieties after infection with *F. solani*.

**Table 2:** The correlation between each replicate of detached leaf and abbreviated stem, above the diagonal indicates the correlation coefficient (r) and below the diagonal showed *p*-value

	Detached Leaf / Abbreviated Stem		
Replicate	I	II	III
I		0.84/0.92	0.89/0.95
II	0.0771/0.0292		0.96/0.91
III	0.0409/0.0132	0.0081/0.0299	

## Discussion

### Infection of detached leaf is a better approach for assessment of DSI upon *F. solani* inoculation

A good pathogeny assessment technology may consider the following features: repeatability, sensitivity, and observation time. Different techniques of *Fusarium* inoculation has been tested in maize ear to find out the best way for evaluating a large number of maize genotypes (Clements et al. 2003). In our study, high correlations detected among all replicates from both organs (Table 2) indicating the trusted repeatability. In addition, the severity pattern from two organs was highly correlated among replicates (Table 1). A similar severity pattern after *Fusarium* inoculation between organs is observed from ear and silk in maize as well (Reid et al. 2002). This finding indicates that the severity rank obtained from the detached leaf well represents the rank detected from the abbreviated stem. Furthermore, the detached leaf showed a wider DSI range among tested varieties than the abbreviated stem, displaying the stronger sensitivity from detached leaf than abbreviated stem. Regarding the observation time, more than 20 days were needed for symptom development after inoculation in the abbreviated stem, similar to a previous study that took 4 weeks for symptom detection in orchids (Su et al. 2012). In contrast, 6 days after inoculation with *F. solani* was enough to distinguish the ranges of symptoms from detached leaf, indicating one-fourth time of inoculation in the abbreviated stem. Last but not least, the space requirement is also an important issue. It was less spacy for the detached leaf as compared to that for the abbreviated stem. A dish with 15 cm diameter and 2 cm height are enough for one sample of the detached leaf, but for the abbreviated stem, a living plant in a 2.5-inch pot is required. From all above mentioned advantages, inoculation in the detached leaf is reliable, more sensitive, shorter time consuming and less experiment space needed than the inoculation in the abbreviated stem, which could be the better solution for *Fusarium* assessment in *Phalaenopsis* orchids.

### Examining the biochemical defense of Phalaenopsis-*Fusarium* interaction

For the defense of the invasion from pathogens, plants develop both structural and biochemical mechanisms to protect themselves. When *Fusarium* spp. infects plants, it stars to destroy the structural defense of cell wall, and then override the plant biochemical defense by producing host-specific mycotoxins (Perincherry et al. 2019). In this study, the first structural defense was avoid since plants were punctured and round wounds were created, which brought the *Fusarium* into the interior part of the plant cells and examined the biochemical defense. In this way, we tested the pathogen-specific host defense directly, and will allow the identification of important factors involved in the defense mechanism for *Fusarium* infection in the future.

## Conclusions

*Phalaenopsis* orchids play the most significant role in flower exportation in Taiwan, but the orchid flower yield has been reduced by the yellow leaf disease caused by *Fusarium* spp. Phenotyping of *Fusarium* symptom is the first step and the most necessary information for further studies such as genome-wide association study. The detached leaf, a better tissue than the original inoculation tissue, the abbreviated stem for inoculation of *Fusarium* isolate “TJP-2178-10” was provided in this study and proved to be with similar distinguish ability, good stability, while shorter time-consuming and lesser space needed. This solution allows a better phenotyping approach and fast detection of yellow leaf disease associated with *Fusarium* for a large number of *Phalaenopsis* samples.

## Data Availability

Not applicable.

## Abbreviations

DSI: Disease severity index
DSL: Disease severity level
QTLs: Quantitative treat loci
